# Self-compassion, self-care, and rhinitis control assessment in patients with allergic rhinitis: a latent profile and mediation analysis

**DOI:** 10.3389/fpubh.2026.1936941

**Published:** 2026-09-14

**Authors:** Xiao Huang, Dongmei Li, Yi Wang, Shi Huang

**Affiliations:** 1 Department of Otorhinolaryngology–Head and Neck Surgery, Clinical Medical College, The First Affiliated Hospital of Chengdu Medical College, Chengdu, China; 2 Department of Hepatobiliary and Vascular Surgery, The First Affiliated Hospital of Chengdu Medical College, Chengdu, Sichuan, China

**Keywords:** allergic rhinitis, person-centered and variable-centered, rhinitis control assessment test, self-care, self-compassion

## Abstract

**Objective:**

This study adopted both variable-centered and person-centered approaches to examine the internal relationships between self-compassion and rhinitis control assessment in patients with allergic rhinitis, and to explore heterogeneity in self-compassion using latent profile analysis.

**Methods:**

From February 2026 to April 2026, 419 patients with allergic rhinitis were recruited from three tertiary Grade A hospitals in Sichuan Province, China. Data were collected using the Self-Compassion Scale, the Rhinitis Control Assessment Test, the Allergic Rhinitis Self-Care Scale, and a demographic information questionnaire.

**Results:**

Self-compassion was significantly and positively associated with rhinitis control (*r* = 0.472) and self-care (*r* = 0.592), and self-care was positively associated with rhinitis control (*r* = 0.693; all *p* < 0.01). In cross-sectional mediation analysis, the indirect association of self-compassion on rhinitis control through self-care was 0.542 [95% confidence interval (CI): (0.431, 0.655)], accounting for 79.09% of the total association [0.685, 95% CI: (0.563, 0.807)]; the direct association was 0.143 [95% CI (0.020, 0.266), 20.91%]. Latent profile analysis identified four self-compassion profiles: the self-kind but low-mindfulness group (10.5%, *n* = 44), the low self-compassion group (9.3%, *n* = 39), the high self-compassion with elevated self-coldness group (70.6%, *n* = 296), and the mindful-but-isolated group (9.5%, *n* = 40). The four profiles did not differ significantly on self-care (*F* = 0.389, *p* = 0.761) or rhinitis control (*F* = 0.915, *p* = 0.434).

**Conclusion:**

In this cross-sectional study of patients with allergic rhinitis, self-compassion and self-care were associated with rhinitis control, and self-care accounted for a substantial proportion of the association between self-compassion and rhinitis control. Four latent self-compassion profiles were identified but did not differ on self-care or rhinitis control. These findings may inform the future longitudinal and intervention research; however, the cross-sectional design does not establish causal pathways, and the effectiveness of profile-based interventions remains to be tested.

## 1 Introduction

Allergic rhinitis is a chronic inflammatory disease of the nasal mucosa mediated by immunoglobulin E (1, 2). A high prevalence, recurrent episodes, and the need for long-term management characterize it (3). Beyond causing physical discomfort, allergic rhinitis can substantially impair learning efficiency, work productivity, social functioning, and emotional wellbeing (4, 5). In recent years, the management of allergic rhinitis has gradually shifted from simple symptom relief toward patient-centered long-term disease control (6). Accordingly, evaluation priorities have expanded from objective inflammatory indicators and pharmacological efficacy to patient-reported outcomes, recovery of daily functioning, and self-management capacity (7). Among available assessment tools, the Rhinitis Control Assessment Test is a concise, practical, and patient-experience-oriented instrument that can effectively reflect recent rhinitis symptoms, disease-related impacts on daily life, and the level of treatment control (8). It therefore provides a basis for clinical evaluation of disease control and optimization of individualized interventions. However, even when receiving similar pharmacological treatments, patients may still show considerable differences in control levels. This suggests that, in addition to disease severity and treatment regimens, psychological resources, behavioral habits, and self-care capacity may play important roles in the control of allergic rhinitis (7). Therefore, this study examined factors associated with disease outcomes in patients with allergic rhinitis from the perspectives of psychological resources, health-related behaviors, and symptom control.

Self-compassion refers to an individual’s tendency to treat oneself with understanding, acceptance, and a nonjudgmental attitude when facing suffering, failure, or illness-related distress (9). Previous studies have shown that self-compassion among patients with chronic diseases can buffer disease-related stress, reduce shame and self-blame, and promote emotional regulation and adaptive coping (10, 11). Patients with allergic rhinitis often experience recurrent symptoms, difficulty fully avoiding triggers, fluctuations in treatment adherence, and impaired quality of life (12). These experiences may give lead to irritability, helplessness, avoidance, or an underestimation of the importance of disease management. Higher levels of self-compassion may help patients interpret symptom fluctuations more gently and acceptably, reduce frustration from recurrent symptoms, and strengthen the psychological foundation for sustained treatment and lifestyle adjustment (13). Thus, self-compassion may not only be directly associated with patients’ subjective evaluations of disease control, but may also indirectly improve rhinitis control by facilitating more adaptive cognitive and emotional responses to the disease.

Self-care in patients with allergic rhinitis refers to the proactive behaviors undertaken in daily life to maintain health and prevent symptom exacerbation, including disease monitoring, symptom recognition, appropriate medication use, and lifestyle adjustment (7, 14). For example, patients with allergic rhinitis may use intranasal corticosteroids, antihistamines, or immunotherapy as prescribed, and avoid allergens such as pollen, dust mites, mold, and animal dander (3, 15). However, the symptoms of allergic rhinitis are often seasonal, intermittent, and easily underestimated (16). Patients may discontinue treatment when symptoms temporarily improve, or develop management fatigue due to long-term recurrent episodes, thereby compromising the continuity and effectiveness of self-care. Self-compassion may enhance the likelihood of engaging in self-care behaviors by strengthening self-care motivation, reducing self-criticism, and promoting rational awareness (17). Individuals with high self-compassion are more likely to perceive health behaviors as a way of caring for their own needs rather than as external pressure or punitive demands. Therefore, when facing treatment inconvenience, environmental constraints, or symptom fluctuations, they may be more likely to remain patient and persistent.

Previous research has confirmed that good self-care is an important foundation for achieving long-term control of allergic rhinitis (18, 19). Control of allergic rhinitis does not depend solely on the prescription of medication, but rather on whether patients can consistently, correctly, and individually implement management strategies in real-life contexts. Standardized medication use can reduce inflammation of the nasal mucosa and alleviate core symptoms such as nasal obstruction, sneezing, and rhinorrhea (20). Trigger identification and environmental control can reduce the frequency of allergen exposure and inflammatory activation (21, 22). Nasal care and healthy lifestyle practices may help improve local mucosal status, sleep quality, and overall perceived health. Conversely, inadequate self-care may lead to irregular treatment, insufficient symptom monitoring, and failure to control triggers (23). Patients may then seek medical care only after symptom exacerbation, forming a cycle of poor disease control and reduced quality of life. As a patient-reported outcome measure, the Rhinitis Control Assessment Test can sensitively reflect patients’ recent symptom burden and perceived disease control, both of which are closely related to daily self-care behaviors. The effect of self-care on disease control is reflected not only at the biomedical level but also at the psycho-behavioral level: active management may enhance patients’ sense of control and illness-mastery beliefs, reduce anxious interpretations of symptom fluctuations, and consequently improve their overall appraisal of disease status. Therefore, self-care may represent an important behavioral pathway linking psychological resources to clinical outcomes.

Based on the above analysis, self-compassion, as an internal psychological resource, may influence how patients understand their illness experiences (24) cope with negative emotions stemming from symptoms and maintain a sense of responsibility for their health during long-term management. Self-care, in turn, may serve as a key behavioral mechanism by which this psychological resource translates into disease-control benefits. When patients experience allergic rhinitis in a more accepting, kind, and mindful manner, they may be more likely to identify symptom changes accurately, proactively adopt preventive and interventional measures, and reduce management interruptions caused by frustration, avoidance, or neglect. Furthermore, improvements in self-care behaviors may enhance rhinitis control by reducing allergen exposure, improving treatment adherence, and promoting healthy lifestyles. Therefore, the effect of self-compassion on rhinitis control may be partially mediated by self-care.

The hypothesized resource → pathway → outcome structure is grounded in the biopsychosocial model of health and illness and, more specifically, in Taylor’s conceptualization of psychosocial resources (25, 26). Within this framework, health-related outcomes emerge from the interplay of biological, psychological, and social processes, and psychosocial resources, such as optimism, mastery, self-esteem, and self-related attitudes, are conceptualized as individual-difference and interpersonal factors that buffer the adverse effects of disease-related stress and promote adaptive coping and, ultimately, mental and physical health. Consistent with this view, self-compassion is positioned here as a psychological resource that shapes how patients appraise and respond to recurrent rhinitis symptoms; self-care is positioned as a behavioral, self-management pathway through which that resource is enacted in daily life; and rhinitis control is positioned as the proximal health-related outcome. This resource → pathway → outcome structure closely parallels the empirical work of Sarid and colleagues on inflammatory bowel disease. In patients with Crohn’s disease and ulcerative colitis, psychological symptoms, economic status, and coping strategies (problem-focused, emotion-focused, and dysfunctional) were differentially associated with active disease (27); dysfunctional coping was associated with poorer satisfaction with life and quality of life, whereas emotion-focused coping and economic status were associated with better outcomes (28); and social support and economic status were shown to reduce psychological distress, which in turn was associated with lower disease activity (29). These studies illustrate precisely the logic applied here: psychological resources and coping/self-management behaviors are measurably linked to disease-related outcomes. Accordingly, the present study applies this established framework to a new clinical population and extends its descriptive reach through a person-centered latent profile approach to characterize heterogeneity in self-compassion.

Although variable-centered analyses of mean-level associations can reveal overall relationships among psychological resources, behavioral management, and disease control in patients with allergic rhinitis, they may obscure important heterogeneity within the patient population. Patients with allergic rhinitis differ in disease duration, allergen exposure, health literacy, and emotional status (30, 31), which may result in distinct latent patterns of self-compassion. Latent profile analysis, as a person-centered approach, enables classification of patients into latent subgroups based on their performance across dimensions of self-compassion, thereby providing a more nuanced understanding of psychological adaptation patterns among patients with allergic rhinitis. This approach can help explain why patients with similar clinical conditions differ in self-care behaviors and rhinitis control, and can inform the development of stratified and precise psycho-behavioral intervention strategies.

Based on the above theoretical rationale, this study proposed the following hypotheses:

*H1*: Self-compassion is significantly and positively associated with rhinitis control assessment in patients with allergic rhinitis.

*H2*: Self-compassion is significantly and positively associated with self-care among patients with allergic rhinitis.

*H3*: Self-care is significantly and positively associated with rhinitis control assessment in patients with allergic rhinitis.

*H4*: Self-care has a cross-sectional mediating effect between self-compassion and rhinitis control assessment in patients with allergic rhinitis.

*H5*: Self-compassion among patients with allergic rhinitis shows significant heterogeneity.

## 2 Methods

### 2.1 Study design

This study used a cross-sectional design to systematically examine the relational structure among self-compassion, self-care, and rhinitis control assessment in patients with allergic rhinitis, as well as the heterogeneous characteristics of the patient population. The study was conducted in strict accordance with the ethical principles of the Declaration of Helsinki. It was reviewed and approved by the Medical Ethics Committee of the First Affiliated Hospital of Chengdu Medical College (No.: 2025CYFYIRB-BA-156). All participants provided written or electronic informed consent. The study was conducted anonymously and confidentially, and no personally identifiable information was collected.

### 2.2 Participants

Recruitment Procedure. A convenience sampling method was used to recruit patients with allergic rhinitis from three tertiary Grade A hospitals in Sichuan Province, China, between February and April 2026. Participants were primarily recruited from otolaryngology and allergy-related outpatient clinics. Researchers first explained the study background, objectives, potential risks, and benefits to potential participants as part of the informed consent process. Patients were invited to complete the questionnaire only after signing written or electronic informed consent. Questionnaires were administered in either paper-based or electronic formats.

To further improve the accuracy of participant selection, the inclusion criteria were as follows: (1) age ≥18 years and ability to independently read and understand Chinese questionnaires; (2) meeting relevant clinical diagnostic criteria for allergic rhinitis, or having been previously diagnosed with allergic rhinitis by specialists in otolaryngology, allergy, or related departments; (3) currently experiencing, or having recurrently experienced within the past year, typical nasal symptoms such as nasal itching, sneezing, watery rhinorrhea, and nasal obstruction; (4) ability to understand the study purpose and questionnaire content, and willingness to provide informed consent.

The exclusion criteria were as follows: (1) comorbid acute upper respiratory tract infection, acute sinusitis, acute exacerbation of nasal polyps, or other acute conditions that might substantially affect the assessment of recent nasal symptoms; (2) history of major nasal surgery with postoperative recovery status that might affect the evaluation of rhinitis symptoms; (3) comorbid severe physical diseases, such as serious cardiac, hepatic, renal, or neurological disorders, or malignant tumors; (4) severe mental disorders, cognitive impairment, disturbance of consciousness, or inability to independently complete the questionnaire; (5) incomplete questionnaire responses, missing key variables, obvious logical inconsistencies, or patterned responses.

The minimum sample size was estimated using G*Power 3.1.9.7 (32). An F test was selected, with the statistical test specified as “Linear multiple regression: Fixed model, R^2^ deviation from zero.” The effect size f^2^ was set at 0.15, α was set at 0.05, and the number of predictors was set at 15. To achieve 95% power, a minimum sample size of 199 participants was required.

The adequacy of the sample for the person-centered analysis was therefore established in three complementary ways. First, Monte Carlo simulation studies indicate that samples of approximately *N* = 300–500 are generally sufficient to recover three to five profiles from six continuous indicators when class separation is moderate to large and mixing proportions are not extremely unbalanced (33); the present sample of 419 participants provided 12.7 observations per free parameter in the retained four-profile model (33 parameters) and at least 39 participants in the smallest profile. Second, the achieved empirical separation was high, the principal determinant of enumeration accuracy in simulation work. Third, a *post hoc* Monte Carlo power analysis was conducted in Mplus following the study by Muthén and Muthén (34): using the parameter estimates of the retained four-profile model as population values, 500 replications with *N* = 419 were generated and analyzed.

During data recruitment, the researchers contacted 463 potential participants. Among them, 24 participants were younger than 18 years, and 7 did not sign informed consent or declined to participate in the questionnaire survey. During data cleaning, 13 questionnaires were excluded due to patterned responses. Therefore, the final valid sample included 419 participants, yielding an effective response rate of 90.49%.

### 2.3 Measures tools

All the analyses in this study used the average values of the core research variables, which ranged from 1 to 5 points.

#### 2.3.1 Self-compassion scale

Self-compassion was assessed using the Self-Compassion Scale developed by Neff (35). The scale contains 26 items covering six dimensions: self-kindness, self-judgment, common humanity, isolation, mindfulness, and over-identification. The scale was translated into Chinese by Chen et al. (36), and its cultural adaptability and reliability were verified in Chinese populations. An example item is, “When I feel emotionally distressed, I try to be more caring toward myself.” In this study, the scale was used to measure self-compassion among Chinese patients with allergic rhinitis. Items were rated on a 5-point Likert scale from 1 = almost never to 5 = almost always. The 13 negatively worded items (the self-judgment, isolation, and over-identification subscales) were reverse scored when computing the overall self-compassion composite. After reverse transformation, the total score ranged from 26 to 130, with higher scores indicating greater overall self-compassion. For the variable-centered analyses, self-compassion was analyzed as the item mean (mean of the 26 items, range 1–5). For the latent profile analysis, the six subscale scores were used as indicators in their original, unreversed orientation, so that higher scores on self-judgment, isolation, and over-identification reflect higher levels of these negative components. In this study, the Cronbach’s alpha coefficient of the Self-Compassion Scale was 0.908, indicating good internal consistency.

#### 2.3.2 Allergic rhinitis self-care questionnaire

Self-care among patients with allergic rhinitis was assessed using items from the disease knowledge and self-care questionnaire for allergic rhinitis patients [Allergic Rhinitis Knowledge and Self-Care (ARKSc) questionnaire] developed by Nizar et al. (37). The self-care section of the questionnaire contains four items and is unidimensional. An example item is, “I attend clinic follow-up visits according to my scheduled appointments.” Items were translated into Chinese, back-translated by bilingual researchers, and reviewed by clinical specialists prior to use. In this study, the questionnaire was used to assess self-care among patients with allergic rhinitis. Items were rated on a 5-point Likert scale from 5 = always to 1 = never. All items were positively scored. The total score ranged from 4 to 20, with higher scores indicating more active self-care behaviors related to allergic rhinitis. The self-care score was analyzed as the item average (mean of the four items, range 1–5). In this study, the Cronbach’s alpha coefficient of the scale was 0.809, indicating good internal consistency. This study employed Analysis of Moment Structures (AMOS) 31.0 software for confirmatory factor analysis. The results indicated that the univariate model had a Good Fit Index [GFI = 0.993, χ^2^/DF = 1.165, Tucker-Lewis Index (TLI) = 0.997, Comparative Fit Index (CFI) = 0.999, Root Mean Square Error of Approximation (RMSEA) = 0.020].

#### 2.3.3 Rhinitis control assessment test

Rhinitis control was assessed using the self-questionnaire for assessing the control of allergic rhinitis developed by Demoly et al. (38). The questionnaire contains five items and is unidimensional. In this study, the questionnaire was used to evaluate allergic rhinitis control, with each item containing five response options. An example item is, “During the past two weeks, have you needed to use additional medication other than that prescribed by your physician to treat your allergic rhinitis?” Response options ranged from 1 = four nights per week or more to 5 = never. All items were positively scored, and the total score ranged from 5 to 25. Higher scores indicated better control of allergic rhinitis. The rhinitis control assessment score was analyzed as the item average (mean of the five items, range 1–5). In this study, the Cronbach’s alpha coefficient of the scale was 0.863, indicating good internal consistency.

### 2.4 Statistical analysis

Statistical analyses were conducted using Statistical Package for the Social Sciences (SPSS) version 27.0, Mplus version 8.3, and the PROCESS macro. First, routine analyses were performed, including reliability testing, common method bias analysis, descriptive statistics, correlation analysis, and between-group comparisons. Next, PROCESS Model 4 was used to test the mediating role of self-care. The mediation effect was examined using bias-corrected bootstrapping with 5,000 resamples. If the 95% confidence interval (CI) for the indirect effect did not include zero, self-care was considered to have a significant mediating effect on the relationship between self-compassion and rhinitis control assessment.

Subsequently, latent profile analysis was conducted to identify latent subgroups of self-compassion among patients with allergic rhinitis. Standardized scores for each dimension of the Self-Compassion Scale were used as profile indicators, and models with one to five classes were fitted sequentially. Model estimation was performed using robust maximum likelihood, with a sufficient number of random starts specified to reduce the risk of converging to local optima. Model selection was based on statistical fit indices, classification quality, and theoretical interpretability. Specifically, we jointly evaluated: (i) elbow plots and the relative (percentage) reduction of Akaike Information Criterion (AIC), Bayesian Information Criterion (BIC), and Sample-Size Adjusted BIC (aBIC), together with the number of additional free parameters required; (ii) the Lo–Mendell–Rubin adjusted likelihood ratio test (LMR-LRT) and the bootstrap likelihood ratio test (BLRT, *p* < 0.05); (iii) classification quality (entropy ≥ 0.80; all average posterior probabilities ≥ 0.70); (iv) class-size adequacy (each class ≥ 5% of the sample and ≥ 30 participants, so that distal-outcome comparisons remained estimable). The most parsimonious model satisfying all criteria was retained. Finally, patients were assigned to latent classes based on their highest posterior probabilities, and the number, proportion, average posterior probability, and self-compassion-dimensional characteristics of each class were reported. Patients were assigned to latent classes based on their highest posterior probability, and between-class differences in distal outcomes (self-care and rhinitis control) were examined using one-way analysis of variance (ANOVA). This most-likely-class-assignment approach does not formally account for classification uncertainty; however, the retained 4-class model showed very high classification precision (entropy = 0.992; average posterior probabilities for the assigned classes ranged from 0.988 to 1.000), which substantially reduces this concern.

Latent profile models with one to five classes were estimated in Mplus version 8.3 using TYPE = MIXTURE with Maximum Likelihood Estimation and Robust standard errors (MLR) via the expectation–maximization algorithm. Within-class indicator variances were estimated as class-invariant and within-class covariances were fixed at zero; a model with class-varying variances was fitted as a sensitivity analysis and did not alter the retained solution. To minimize the risk of local maxima, 250 random sets of starting values were requested, with the 50 best solutions retained for final-stage optimization (STARTS = 250 50). Convergence criteria were the Mplus defaults for mixture models, that is, a relative log-likelihood convergence criterion of 1 × 10^−6^ and an observed-data log-likelihood derivative criterion of 1 × 10^−6^.

Standardized scores for each of the six dimensions of the Self-Compassion Scale were used as profile indicators. The three negatively valanced dimensions were entered in their original, unreversed orientation, such that higher values indicate higher levels of these negative components; the reverse-scoring described was applied only when computing the overall self-compassion composite used in the variable-centered analyses.

## 3 Results

### 3.1 Demographic characteristics

A total of 419 patients with allergic rhinitis were included in this study, with a mean age of 39.86 ± 11.28 years. Among them, 217 participants were men (51.8%) and 202 were women (48.2%). Overall, the sample was predominantly composed of married participants (45.8%), individuals with a bachelor’s degree or above (51.3%), and urban residents (68.3%). Detailed demographic characteristics are presented in Table 1.

**Table 1:** Summary of demographic characteristics.

Variables	Items	Number (N)	Proportion (%)
Gender	Male	217	51.8
	Female	202	48.2
Marital status	Unmarried	149	35.6
	Married	192	45.8
	Divorced	47	11.2
	Widowed spouse	31	7.4
Educational background	Primary school and below	66	15.8
	Middle school or high school	138	32.9
	Bachelor’s degree or above	215	51.3
Place of residence	Cities	286	68.3
	Countryside	133	31.7
Occupational status	Student	26	6.2
	Worker	305	72.8
	Unemployment	57	13.6
	Retirement	31	7.4
Monthly income level	≤4.000 Renminbi (RMB)	90	21.5
	4,001–8,000 RMB	194	46.3
	≥8,001 RMB	135	32.2
Course of allergic rhinitis	≤1 year	85	20.3
	1–5 years	139	33.2
	6–10 years	110	26.3
	≥10 years	85	20.3
Types of rhinitis	Seasonality	167	39.9
	perennial	252	60.1
Smoking	Yes	171	40.8
	No	248	59.2
Drinking alcohol	Yes	179	42.7
	No	240	57.3
Heredity	Yes	171	40.8
	No	248	59.2
Age	Years	39.86 ± 11.28	

### 3.2 Common method bias test

Although this study adopted several procedural measures to reduce common method bias, including anonymous participation, confidentiality assurances, and reverse-scored items, the data were primarily collected through patient self-report questionnaires and may still be subject to bias. To examine whether common method bias may have affected the study findings, Harman’s single-factor test was conducted. The results showed that the unrotated exploratory factor analysis extracted five factors with eigenvalues greater than 1. The first factor explained 28.622% of the total variance, which was below the critical threshold of 40%. These findings indicate that serious common method bias was unlikely to be present in this study.

### 3.3 Descriptive statistics and correlation analysis

Descriptive statistics and correlation analyses were performed for the core study variables, as shown in Table 2. The results showed that the mean score for self-compassion was 3.763 ± 0.608, the mean score for self-care among patients with allergic rhinitis was 3.715 ± 0.813, and the mean score for the Rhinitis Control Assessment Test was 3.764 ± 0.875. The absolute values of skewness for the main variables ranged from 0.979 to 1.470, and the absolute values of kurtosis ranged from 0.222 to 1.807. According to the criteria proposed by Kline (39), these values were within acceptable ranges, suggesting that the main variables were approximately normally distributed.

**Table 2:** Descriptive statistics and correlation analysis of core study variables.

Variables	*M*	SD	Skewness	Kurtosis	1	2	3
Self-compassion	3.763	0.608	−1.470	1.807	1		
Self-care	3.715	0.813	−0.979	0.222	0.592**	1	
Rhinitis control assessment	3.764	0.875	−1.368	0.767	0.472**	0.693**	1

**p < 0.01. SD, standard deviation.

The correlation analysis showed that self-compassion was significantly and positively correlated with self-care among patients with allergic rhinitis (*r* = 0.592, *p* < 0.01). Self-compassion was also significantly and positively correlated with the Rhinitis Control Assessment Test score (*r* = 0.472, *p* < 0.01). In addition, self-care among patients with allergic rhinitis was significantly and positively correlated with the Rhinitis Control Assessment Test score (*r* = 0.693, *p* < 0.01).

### 3.4 Between-group differences in rhinitis control assessment scores

To further examine differences in Rhinitis Control Assessment scores among patients with varying demographic and disease-related characteristics, independent-samples *t*-tests and one-way analysis of variance were conducted, as shown in Table 3. The results indicated no significant differences in Rhinitis Control Assessment scores by gender, marital status, educational level, place of residence, occupational status, disease duration of allergic rhinitis, type of rhinitis, smoking status, alcohol consumption, or hereditary factors (*p* > 0.05). Notably, Rhinitis Control Assessment scores differed significantly across monthly income levels (*F* = 4.980, *p* = 0.007). The ≤4,000 Renminb (RMB) group differed significantly from the ≥8,001 RMB group (*p* = 0.008), and from the 4,001–8,000 RMB group (*p* = 0.029); no significant difference was found between the 4,001–8,000 RMB and ≥8,001 RMB groups (*p* > 0.05).

**Table 3:** Between-group analysis of rhinitis control assessment scores.

Variables	Items	*M*	SD	t/F	*p*	Cohen d/η^2^
Gender	Male	3.747	0.881	−0.428	0.669	−0.042	Female	3.783	0.871			
	Marital status	Unmarried	3.732	0.897	0.364	0.779	0.003	Married	3.771	0.867				Divorced	3.745	0.855				Widowed spouse	3.910	0.878			
Educational background		Primary school and below	3.736	0.869	0.188	0.829	0.001	Middle school - High school	3.738	0.928				Bachelor degree or above	3.790	0.845			
		Place of residence	Cities	3.729	0.899	−1.219	0.223	−0.128	Countryside	3.841	0.821			
			Occupational status	Student	3.608	0.974	1.985	0.116	0.014	Worker	3.800	0.849				Unemployment	3.551	1.002				Retirement	3.935	0.742			
Monthly income level	≤4,000 RMB ^a^	3.516		1.012	4.980	0.007	0.023	4,001–8,000 RMB ^b^	3.803	0.869				≥8,001 RMB ^b^	3.874	0.752			
	Course of allergic rhinitis	≤1 year		3.736	0.896	0.429	0.733	0.003	1–5 years	3.738	0.897				6–10 years	3.845	0.797				≥10 years	3.729	0.924			
		Types of rhinitis		Seasonality	3.766	0.858	0.043	0.966	0.004	Perennial	3.763	0.888			
Smoking			Yes	3.737	0.869	−0.531	0.596	−0.053	No	3.783	0.881			
		Drinking alcohol	Yes	3.678	0.943	−1.741	0.082	−0.172	No	3.828	0.817			
Heredity	Yes		3.780	0.862	0.309	0.758	0.031	No	3.753	0.886			

M, mean; SD, standard deviation. Different subscript letters in the same column indicate significant differences between groups after Bonferroni correction. Groups sharing the same letter are not significantly different.

### 3.5 Cross-sectional mediation analysis of self-care among patients with allergic rhinitis

To examine the mediating role of self-care in the relationship between self-compassion and Rhinitis Control Assessment scores among patients with allergic rhinitis, a cross-sectional mediation analysis was performed using Model 4 of the PROCESS macro. The bootstrap method with 5,000 resamples was used to estimate the 95% confidence interval of the indirect effect. In the model, self-compassion was entered as the independent variable, the Rhinitis Control Assessment score as the dependent variable, and self-care among patients with allergic rhinitis as the mediator. Meanwhile, based on previous findings, monthly income level, sex, disease duration of allergic rhinitis, type of allergic rhinitis, smoking status, alcohol consumption, and genetic/family history factors were included as control variables.

As shown in Table 4 and Figure 1, in Model 1, self-compassion has a significant and positive association with self-care among patients with allergic rhinitis [*B* = 0.791, *p* < 0.001, 95% CI = (0.687, 0.894)]. The coefficient of determination for Model 1 was *R*^2^ = 0.372, and the overall model was significant (*F* = 30.400), indicating that the model (self-compassion together with the covariates) explained 37.2% of the variance in self-care among patients with allergic rhinitis.

**Table 4:** Linear regression analysis of self-care among patients with allergic rhinitis.

Model	Model 1					Model 2				
Predictor	Self-care					Rhinitis control assessment				
	*B*	SE	β	*t*	95%CI	*B*	SE	β	*t*	95% CI
Self-compassion	0.791	0.053	0.591	14.999***	[0.687, 0.894]	0.143	0.063	0.099	2.284*	[0.020, 0.266]
Self-care						0.685	0.047	0.636	14.521***	[0.592, 0.778]
*R*	0.610					0.712				
*R* ^2^	0.372					0.507				
*F*	30.400***					46.750***				

*p < 0.05; ***p < 0.001.

**Figure 1:**
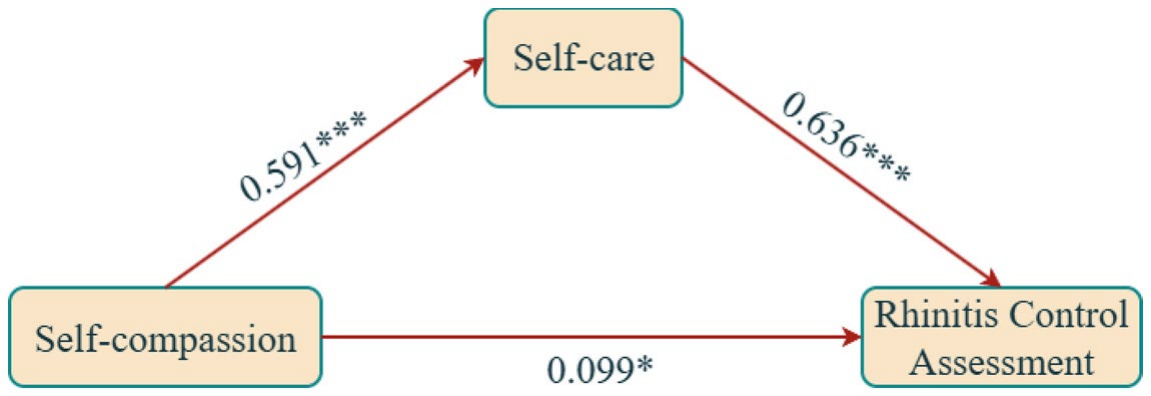
Cross-sectional mediation path coefficients for self-care. *p < 0.05; ***p < 0.001.

In Model 2, self-care was significantly and positively associated with Rhinitis Control Assessment scores [*B* = 0.685, SE = 0.047, *β* = 0.636, *t* = 14.521, *p* < 0.001, 95% CI = (0.592, 0.778)]. The cross-sectional direct association of self-compassion with Rhinitis Control Assessment scores remained statistically significant [*B* = 0.143, SE = 0.063, *β* = 0.099, *t* = 2.284, *p* = 0.023, 95% CI = (0.020, 0.266)]. The coefficient of determination for Model 2 was *R*^2^ = 0.507, and the overall model was significant (*F* = 46.750), indicating that the model explained 50.7% of the variance in Rhinitis Control Assessment Test scores.

The bootstrap mediation analysis results are presented in Table 5. The total cross-sectional associations of self-compassion on Rhinitis Control Assessment scores were 0.685 [standard error (SE) = 0.062, 95% CI = (0.563, 0.807)]. The statistical direct effects of self-compassion on Rhinitis Control Assessment scores were 0.143 [SE = 0.063, 95% CI = (0.020, 0.266)], accounting for 20.91% of the total effect. The statistical indirect effects through the pathway of self-compassion → self-care among patients with allergic rhinitis → Rhinitis Control Assessment scores were 0.542 [SE = 0.058, 95% CI = (0.431, 0.655)]. As the 95% confidence interval of the indirect effect did not include zero, self-care among patients with allergic rhinitis was found to exert a significant partial cross-sectional mediating effect between self-compassion and Rhinitis Control Assessment scores, supporting H4.

**Table 5:** Bootstrap cross-sectional mediation effect of self-care among patients with allergic rhinitis.

Effect of type	Effect	SE	LLCI	ULCI	Effect proportion (%)
Total effect	0.685	0.062	0.563	0.807	100.00
Direct effect	0.143	0.063	0.020	0.266	20.91
Indirect effect	0.542	0.058	0.431	0.655	79.09

LLCI, lower level (or limit) confidence interval; ULCI, upper level (or limit) confidence interval.

### 3.6 Latent profile analysis of self-compassion

In this study, standardized scores for each dimension of the Self-Compassion Scale were used as profile indicators, and latent profile analysis was conducted using Mplus version 8.3. Models with 1–5 latent classes were fitted sequentially. The model fit indices are presented in Table 6. The results showed that as the number of latent classes increased from 1 to 5, the AIC, BIC, and aBIC values gradually decreased, indicating progressive improvement in model fit. The entropy values for the 2–5-class models were all above 0.80, indicating good classification accuracy across models. The likelihood ratio tests showed that the LMR tests for the 2–5-class models were all statistically significant. The BLRT tests were also statistically significant, indicating that adding one latent class significantly improved model fit compared with the model containing one fewer class.

**Table 6:** Model fit indices for latent profile analysis.

Index	Profile 1	Profile 2	Profile 3	Profile 4	Profile 5
Loglikelihood	−3162.1	−2658.664	−2529.004	**−2315.991**	−2232.056
Free parameters	12	19	26	**33**	40
AIC	6348.200	5355.327	5110.008	**4697.983**	4544.112
BIC	6396.655	5432.047	5214.993	**4831.232**	4705.627
aBIC	6358.575	5371.754	5132.487	**4726.514**	4578.695
Entropy	–	0.995	0.984	**0.992**	0.985
LMR (p)	–	<0.001	0.007	**<0.001**	0.048
BLRT (p)	–	<0.001	<0.001	**<0.001**	<0.001
Class 1	–	19.81%	8.59%	**10.50%**	8.83%
Class 2	–	80.19%	11.22%	**9.31%**	10.98%
Class 3	–	–	80.19%	**70.64%**	10.74%
Class 4	–	–	–	**9.55%**	62.05%
Class 5	–	–	–	–	7.40%

The bolded parts represent the indicators of the optimal potential profile.

The 5-class solution differed from the 4-class solution mainly by splitting the dominant class of the 4-class model (70.6%, *n* = 296) into a large class (62.1%) and a small additional class of 31 participants (7.4%). Class 5 was substantively redundant rather than qualitatively distinct: its six subscale means paralleled the shape of the adjacent large class and differed only in elevation, and it did not correspond to a theoretically meaningful or clinically actionable configuration. In addition, Class 5 lay just above the pre-specified minimum class size (≥30 participants and ≥5% of the sample), the LMR-LRT was only marginally significant (*p* = 0.048), and the reduction in BIC associated with adding the fifth class (ΔBIC = 125.61) was approximately one third of that observed when adding the fourth class (ΔBIC = 383.76), indicating that the improvement in fit had plateaued. The BLRT was significant for every adjacent comparison (all *p* < 0.001) and therefore provided no basis for discriminating between the 4- and 5-class solutions. As the 5-class model did not yield a distinct, stable, and interpretable profile, the 4-class model was retained.

Therefore, the 4-class model was ultimately selected as the optimal latent profile model of self-compassion among patients with allergic rhinitis, as shown in Table 7.

**Table 7:** Absolute and relative change in information criteria across adjacent latent profile models.

Comparison	ΔLL	Δ free parameters	ΔAIC	ΔBIC	ΔaBIC	Smallest class (n)	LMR (p)
1 → 2	503.44	7	992.87	964.61	986.82	83	<0.001
2 → 3	129.66	7	245.32	217.05	239.27	36	0.007
3 → 4	213.01	7	412.03	383.76	405.97	39	<0.001
4 → 5	83.94	7	153.87	125.61	147.82	31	0.048

Based on the classification results of the 4-class model, a profile plot was generated using Origin 2026, as shown in Figure 2. Class 1 showed relatively high scores on the dimensions of self-kindness and common humanity, but also high levels of self-judgment and relatively low levels of mindfulness. This profile reflected the coexistence of positive self-acceptance and negative self-evaluation and was therefore labeled the self-kind but low-mindfulness group. Class 2 showed generally low scores across all six dimensions, particularly on the positive dimensions of self-kindness, common humanity, and mindfulness. This profile was therefore labeled the low self-compassion group. Class 3 showed overall high scores across the dimensions of self-compassion. Patients in this class exhibited relatively high levels of self-kindness, common humanity, and mindfulness, while also showing high levels of self-judgment, isolation, and over-identification. This pattern suggested that these patients had highly active psychological responses and were therefore labeled as high self-compassion with elevated self-coldness. Class 4 showed high scores on self-kindness and mindfulness, but also relatively high levels of isolation and over-identification. Meanwhile, scores on common humanity and self-judgment were relatively low. This profile reflected the coexistence of mindful awareness and isolated, illness-related experiences and was therefore labeled the mindful-but-isolated self-compassion group (Table 8).

**Figure 2:**
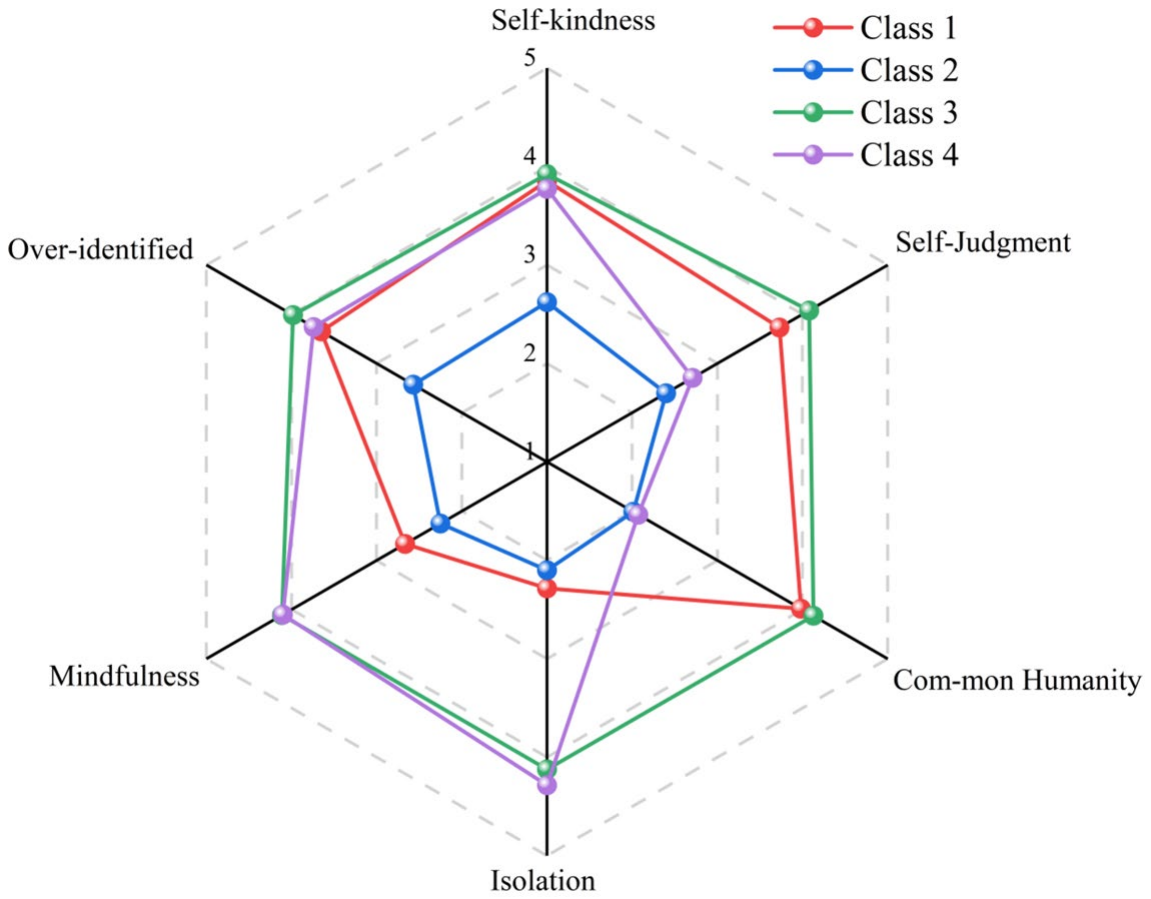
A profile plot of the optimal latent profile. Class 1: Self-kind but low-mindfulness; Class 2: Low self-compassion; Class 3: High self-compassion with elevated self-coldness; Class 4: Mindful-but-isolated self-compassion.

**Table 8:** Average posterior probabilities of the optimal latent profile model.

Class	Class 1	Class 2	Class 3	Class4
Class 1	0.988	0.011	0.001	0.000
Class 2	0.000	1.000	0.000	0.000
Class 3	0.002	0.000	0.998	0.000
Class 4	0.000	0.000	0.009	0.991

The one-way analysis of variance (ANOVA) showed no statistically significant differences among the four categories in either the self-care (*F* = 0.389, *p* = 0.761) or the rhinitis control assessment (*F* = 0.915, *p* = 0.434), as shown in Table 9.

**Table 9:** The optimal latent profile in self-care and rhinitis control assessment was analyzed by ANOVA.

Class	*N* (%)	Self-care M (SD)	Rhinitis control assessment (SD)
Class 1: self-kind but low-mindfulness	44 (10.5%)	3.76 (0.94)	3.65 (1.05)
Class 2: low self-compassion	39 (9.3%)	3.76 (0.63)	3.71 (0.91)
Class 3: high self-compassion with elevated self-coldness	296 (70.6%)	3.69 (0.82)	3.76 (0.86)
Class 4: mindful-but-isolated self-compassion	40 (9.5%)	3.81 (0.77)	3.95 (0.72)
*F*		0.389	0.915
*P*		0.761	0.434

## 4 Discussion

### 4.1 Variable-centered analysis: relationships among self-compassion, rhinitis control assessment, and self-care

The variable-centered analysis further revealed the association pathways among self-compassion, Rhinitis Control Assessment scores, and self-care. As a positive psychological resource, self-compassion enables individuals to respond to their own condition with understanding and acceptance when facing recurrent sneezing (40), nasal congestion, rhinorrhea, nasal itching, sleep disturbance, or impairment in work and study, rather than excessive self-blame, shame, or avoidance. This psychological trait may promote self-care behaviors through multiple mechanisms.

First, self-compassion may help reduce disease-related self-blame and negative emotional responses (41, 42). The recurrent, chronic, and unpredictable nature of allergic rhinitis often causes patients to experience irritability, frustration, helplessness, and even shame (43). For example, patients may worry that frequent nose blowing, sneezing, or reduced concentration will affect their social interactions and work performance (4). Patients with low self-compassion are more likely to fall into self-criticism and emotional amplification when facing these difficulties (44), thereby weakening the psychological energy required for active disease management. By contrast, patients with high self-compassion and elevated self-kindness are more likely to understand symptom fluctuations as part of the natural course of the disease and to seek constructive solutions, such as standardized medication use, lifestyle adjustment, or active consultation with healthcare professionals.

Second, self-compassion may enhance patients’ motivation to engage in health behaviors (45). Self-compassion is a positive form of self-regulation based on caring for one’s own health (46). When patients treat themselves with kindness rather than harsh criticism, they are more likely to regard self-care as an act of self-care. This motivational shift is particularly important for chronic disease management. Daily care for allergic rhinitis often involves repetitive behaviors, such as regular medication use, nasal irrigation, bedroom cleaning, and reducing allergen exposure (47). If patients rely mainly on short-term symptom pressure to maintain self-care behaviors, they may easily discontinue these behaviors once symptoms are relieved. In contrast, health motivation based on intrinsic self-care and concern is more likely to support behavioral persistence.

The cross-sectional analysis further supported a pattern of association consistent with self-care as an intervening variable in the relationship between self-compassion and rhinitis control. As all variables were assessed at a single time point, these findings cannot establish temporal ordering or causation; the indirect association is best interpreted as evidence that self-compassion, self-care, and rhinitis control covary in a manner consistent with a hypothesized mediational structure, not as proof that self-compassion leads to improved self-care or that self-care subsequently improves rhinitis control. Patients with higher self-compassion were more likely to develop a positive, stable, and non-defensive attitude toward disease management, thereby contributing to better rhinitis control. If the observed associations reflect causal processes, psychological resources and disease control could form a mutually reinforcing cycle: self-compassion may promote adaptive management, adaptive management may improve symptom control, and symptom improvement may in turn strengthen patients’ confidence and willingness to continue self-care. Longitudinal research is required to test this possibility. Conversely, low self-compassion, poor disease control, and inadequate self-care may also form a negative cycle, gradually leading patients to experience helplessness and management fatigue during repeated symptom episodes.

The person-centered results complemented the variable-centered results. Latent profile analysis answered the question of “what different self-care/self-compassion patterns exist among patients.” In contrast, mediation analysis answered the question of “how psychological resources and disease control are associated with self-care.” Combining these two perspectives provides a more comprehensive understanding of self-care among patients with allergic rhinitis. On the one hand, patients can be identified as distinct risk subgroups based on their psychological or behavioral patterns. On the other hand, self-compassion and rhinitis control may be important psychological and clinical factors explaining differences across these subgroups. This integrated perspective may help shift allergic rhinitis management from symptom control alone to an approach that integrates psychological resources, health behavior, and disease control.

### 4.2 Person-centered analysis: heterogeneity of self-compassion among patients with allergic rhinitis

Self-compassion among patients with allergic rhinitis consists of multiple dimensions, including self-kindness, self-judgment, common humanity, isolation, mindfulness, and over-identification (35), which may combine in different ways. In this study, four latent profiles were identified and labeled as the self-kind but low-mindfulness group, low self-compassion group, high self-compassion with elevated self-coldness group, and mindful-but-isolated self-compassion group. These findings suggest that, in the context of recurrent chronic rhinitis symptoms and persistent disease burden, patients’ psychological responses to their illness experiences are complex and diverse.

Class 1 was labeled the self-kind but low-mindfulness group. Patients in this group scored relatively high on self-kindness and common humanity, indicating that they were able, to some extent, to treat their illness experience with a gentle, accepting attitude and to recognize that symptom-related distress was not unique to them but rather a common experience among patients with chronic illness. However, this group also showed relatively high self-judgment and lower mindfulness, suggesting the coexistence of positive self-acceptance and negative self-evaluation. This pattern could be interpreted as reflecting patients who cognitively understand the objective nature of disease recurrence and are willing to treat themselves kindly, yet may still attribute poor disease control to insufficient self-management or a lack of willpower, which may lead to self-blame and guilt. These mechanisms remain to be tested in the future studies. Due to their relatively low level of mindfulness, these patients may find it difficult to observe symptoms and emotional changes in a non-judgmental manner stably. They may easily fall into repetitive thinking and negative evaluation when symptoms worsen. Therefore, this group does not represent high self-compassion in a true sense, but rather a transitional state in which psychological resources and psychological vulnerability coexist.

Class 2 was labeled the low self-compassion group. This group showed insufficient levels of positive dimensions such as self-kindness, common humanity, and mindfulness, indicating a lack of stable self-care capacity, recognition of shared illness experiences, and non-judgmental awareness. Patients in the low self-compassion group may find it more difficult to regard allergic rhinitis as a chronic health problem requiring long-term management. They may be more likely to adopt passive, avoidant, or short-term coping strategies when symptoms occur. Due to insufficient positive psychological resources, patients may lack both understanding and tolerance toward their own situation and the psychological motivation to adjust their behaviors when facing recurrent nasal symptoms actively.

It is worth noting that this group also did not score highly on negative dimensions such as self-judgment, isolation, and over-identification. These patients may neither strongly criticize themselves nor sufficiently care for themselves; they may neither be deeply immersed in symptom-related distress nor demonstrate adequate mindful awareness of symptoms and emotional experiences. This low-activation state may lead to insufficient awareness of the importance of disease management, especially during symptom remission, when patients may be more likely to neglect continuous care and preventive management.

Class 3 was labeled the high self-compassion with elevated self-coldness group. This group showed generally high scores across all six dimensions of self-compassion. Among some patients, positive and negative dimensions may increase simultaneously, forming a pattern of highly active psychological responses. These patients may have strong awareness of their illness experience and be willing to understand their situation with a kind attitude. Still, they may also be highly sensitive to symptom changes, disease impact, and self-management outcomes, making them prone to self-evaluation, feelings of loneliness, and repetitive rumination.

The recurrent and unpredictable nature of allergic rhinitis symptoms may cause these patients to continuously monitor bodily changes and experience strong emotional reactions with each symptom fluctuation. On the one hand, higher mindfulness and self-kindness may help patients actively engage in self-care behaviors. On the other hand, higher over-identification and isolation may also cause them to pay excessive attention to symptom details and interpret temporary discomfort as a signal of disease-control failure, thereby increasing psychological exhaustion. Therefore, patients in this group may not lack self-care awareness; rather, they may face the challenge of transforming heightened awareness into stable, moderate, and effective disease management.

Class 4 was labeled the mindful-but-isolated self-compassion group. Patients in this group scored relatively high on self-kindness and mindfulness, indicating that they were able to perceive their symptoms and emotions to some extent and had a relatively kind attitude toward themselves. However, this group also showed relatively high levels of isolation and over-identification, while scores on common humanity and self-judgment were relatively low. This pattern reflected the coexistence of mindful awareness and isolated illness-related experiences.

Mindfulness is generally regarded as an important positive component of self-compassion. However, the present study found that high mindfulness does not necessarily imply low isolation or low over-identification. If mindful awareness lacks the support of common humanity, patients may simply perceive their symptoms and emotions more clearly but may not necessarily experience reduced isolation. The sleep disturbance, daytime fatigue, reduced attention, social embarrassment, and long-term medication burden experienced by patients with allergic rhinitis may be highly subjective. When patients lack emotional validation from family members, peers, or healthcare professionals, they may maintain a high level of isolation and symptom-related immersion even when they possess relatively high mindfulness.

### 4.3 Implications and directions for the future intervention research

The profile-specific intervention strategies outlined below are suggestions grounded in the descriptive profiles described above. As these strategies were not tested in the present cross-sectional study, they should be regarded as directions for the future intervention research rather than as evidence-based clinical recommendations.

This study emphasizes that the management of patients with allergic rhinitis should incorporate the assessment of self-care ability into routine nursing and clinical care. A single outpatient visit is often insufficient to cover the full range of management needs in patients’ daily lives. By assessing patients’ behavioral performance in medication use, environmental control, symptom monitoring, and psychological adjustment, healthcare professionals can more accurately identify weaknesses in disease management and develop individualized care plans.

For patients in the self-kind but low-mindfulness group, nursing interventions should focus on the coexistence of positive acceptance and self-criticism. Clinical nurses may use brief mindfulness exercises, symptom-record feedback, cognitive restructuring, and positive self-talk training to help these patients recognize the objective nature of disease recurrence, reduce unreasonable self-blame, and view symptom changes in a more objective and non-judgmental manner.

For patients in the low self-compassion group, interventions should focus on enhancing disease-management awareness and stimulating motivation for self-care. Healthcare professionals should strengthen health education for these patients and help them understand the chronic and recurrent nature of allergic rhinitis, as well as the importance of standardized management. Practical and concrete behavioral guidance may be particularly useful, such as establishing medication reminders, simplifying nasal irrigation procedures, developing allergen-avoidance checklists, and setting gradual self-care goals.

For patients in the high self-compassion with elevated self-coldness group, the intervention focus should be on reducing excessive psychological involvement and promoting balance between moderate awareness and effective action. Healthcare professionals should help these patients establish a management pattern based on appropriate monitoring and reasonable coping. Patients may be guided to use structured symptom records, limit the amount of time spent focusing on symptoms each day, and reduce repetitive checking and excessive worry. At the same time, relaxation training, emotion regulation, and problem-solving skills can be combined to help patients transform heightened attention to illness into specific and feasible self-care behaviors.

For patients in the mindful-but-isolated self-compassion group, interventions should focus on strengthening perceived social support and common humanity. Healthcare professionals should provide more emotional validation during health education and follow-up, actively ask about the specific effects of symptoms on daily life, and avoid simplifying or minimizing the disease experience. Peer education, patient support groups, family participation, and continuous follow-up may help patients recognize that their illness-related difficulties are understandable and shared by others, thereby reducing isolation and improving engagement in self-care.

The Rhinitis Control Assessment Test may serve as an important tool linking psychological assessment and clinical decision-making. Rhinitis control not only reflects symptom severity but may also influence patients’ judgments about the effectiveness of self-care. For patients with poor rhinitis control, healthcare professionals should avoid simply attributing the problem to non-adherence or lack of attention. Instead, they should further assess whether there are medication technique errors, continuous allergen exposure, comorbid sinusitis or asthma, insufficient treatment regimens, or excessive psychological stress. By combining rhinitis control assessment with self-care or self-compassion profiles, clinicians may more precisely distinguish patients with insufficient ability, insufficient motivation, excessive symptom burden, or insufficient psychological resources, thereby improving the matching of interventions.

### 4.4 Limitations and the future directions

Despite the above findings, several limitations should be acknowledged. First, this study adopted a cross-sectional design, which prevents causal inference among variables. There may be bidirectional or even cyclical relationships among self-compassion, rhinitis control, and self-care. For example, better self-care may improve rhinitis control, and good disease control may in turn enhance patients’ self-efficacy and positive self-attitudes. Conversely, long-term poor symptom control may weaken patients’ psychological resources and lead to reduced self-compassion. Therefore, the future studies should adopt longitudinal designs and repeatedly measure relevant variables across seasons, symptom-fluctuation stages, and treatment time points to reveal their temporal relationships.

Second, the majority of the variables in this study relied on patient self-reports, which may be affected by recall bias, social desirability bias, and common method bias. Patients may overestimate their medication adherence or environmental control behaviors, or they may evaluate their overall self-care more negatively because of recent severe symptoms. The future studies could combine multi-source data to improve measurement accuracy, such as electronic medication monitoring, prescription refill records, nasal irrigation frequency records, objective indicators of allergen exposure, physician assessments, nasal physical examinations, and mobile-based real-time symptom diaries. Combining subjective reports with objective indicators would help more comprehensively characterize patients’ self-care behaviors and disease-control status.

Third, although latent profile analysis can reveal population heterogeneity, the number and naming of classes depend to some extent on statistical indicators, theoretical interpretation, and sample characteristics. Patients from different regions, age groups, disease severities, or healthcare settings may present different profile structures. Therefore, the future studies should assess whether the latent classes identified in this study remain stable in larger samples, multicenter settings, and populations with different cultural backgrounds. In addition, latent transition analysis may be used to explore transitions among different self-care or self-compassion classes over time and to identify factors that promote movement from low self-care or maladaptive psychological states toward higher self-care or more adaptive states.

Fourth, this study may not have fully included important external factors affecting self-care. The management of allergic rhinitis depends not only on individual psychological resources but also on multiple factors, such as social support, family environment, economic burden, healthcare accessibility, health literacy, quality of doctor–patient communication, work and study stress, air pollution, and allergen exposure. Several clinically relevant confounders, including current pharmacotherapy, medication adherence, disease severity, asthma or sinusitis comorbidity, and allergen exposure, were not measured; residual confounding therefore cannot be excluded. The future research should construct a more comprehensive ecological model to examine the interactions among individual, family, healthcare system, and environmental factors. For example, the future studies may explore whether social support buffers the adverse effects of low self-compassion on self-care, whether health literacy moderates the relationship between rhinitis control and self-care, and whether seasonal allergen exposure alters the stability of patients’ self-care or self-compassion profiles. These issues warrant further investigation.

In addition, between-class differences in self-care and rhinitis control were tested after most-likely class assignment followed by ANOVA, which does not formally propagate classification error. Although the high posterior probabilities (0.988–1.000) mitigate this concern, the future studies should employ the three-step [e.g., Bolck, Croon, and Hagenaars (BCH)] or classify–analyze approaches that adjust for classification uncertainty.

## 5 Conclusion

In this cross-sectional study of 419 patients with allergic rhinitis, self-compassion and self-care were positively associated with rhinitis control, and self-care showed a significant cross-sectional indirect association between self-compassion and rhinitis control. Four distinct self-compassion profiles were identified, but they did not differ in self-care or rhinitis control. These findings may inform the future longitudinal studies and the development of stratified interventions; however, the cross-sectional design does not permit causal inference, and the effectiveness of profile-based interventions was not demonstrated. The present study applies an established biopsychosocial, psychosocial-resources framework to a new clinical population rather than extending that theory; its primary methodological extension is the person-centered characterization of heterogeneity in self-compassion.

## Data Availability

The raw data supporting the conclusions of this article will be made available by the authors, without undue reservation.
